# COMplementary Primer ASymmetric PCR (COMPAS-PCR) Applied to the Identification of *Salmo salar*, *Salmo trutta* and Their Hybrids

**DOI:** 10.1371/journal.pone.0165468

**Published:** 2016-10-26

**Authors:** Marc B. Anglès d’Auriac

**Affiliations:** Department of Aquaculture, Norwegian Institute for Water Research, Oslo, 0349, Norway; Oklahoma State University, UNITED STATES

## Abstract

Avoiding complementarity between primers when designing a PCR assay constitutes a central rule strongly anchored in the mind of the molecular scientist. 3’-complementarity will extend the primers during PCR elongation using one another as template, consequently disabling further possible involvement in traditional target amplification. However, a 5’-complementarity will leave the primers unchanged during PCR cycles, albeit sequestered to one another, therefore also suppressing target amplification. We show that 5’-complementarity between primers may be exploited in a new PCR method called COMplementary-Primer-Asymmetric (COMPAS)-PCR, using asymmetric primer concentrations to achieve target PCR amplification. Moreover, such a design may paradoxically reduce spurious non-target amplification by actively sequestering the limiting primer. The general principles were demonstrated using 5S rDNA direct repeats as target sequences to design a species-specific assay for identifying *Salmo salar* and *Salmo trutta* using almost fully complementary primers overlapping the same target sequence. Specificity was enhanced by using 3’-penultimate point mutations and the assay was further developed to enable identification of *S*. *salar* x *S*. *trutta* hybrids by High Resolution Melt analysis in a 35 min one-tube assay. This small paradigm shift, using highly complementary primers for PCR, should help develop robust assays that previously would not be considered.

## Introduction

Since the Polymerase Chain Reaction (PCR) was invented in the mid-80s [1], Nucleic Acid amplification techniques have had an unprecedented development for molecular biology applications. A contributing factor to this success is its flexibility with the development of several modifications which expands the technical capabilities of PCR. For example, several methods have been developed for the detection of point mutations such as the Amplification Refractory Mutation System (ARMS) [2] and variants such as the PCR Amplification of Specific Alleles (PASA) [3, 4], bidirectional-PASA [5] or Mismatch Amplification Mutation Assay (MAMA) [6], Taq-MAMA [7] and Melt-MAMA [8]. Detection of point mutations, also called Single Nucleotide Polymorphism (SNP) and Single Nucleotide Variants (SNV), may be relevant in various contexts spanning from medical applications for infectious disease, genetic and cancer diagnostics to population genetics and species identification. However, limitations inherent to DNA chemistry may reduce PCR specificity or sensitivity. For instance, non-target amplification and in particular primer complementarity leading to Primer Dimer (PD) formation is a well-known limiting factor for the design of PCR assays. PD formation may considerably reduce PCR reaction efficiency and is carefully avoided when designing an assay i.e. by using ad hoc software packages [9]. A 3’ complementarity between primers may be detrimental during PCR as annealing between the primers will elicit DNA extension by the DNA polymerase, producing a non-target amplicon itself being a perfect match for further non-target amplification competing with target amplification. Moreover, it has emerged that PD formation appears to be incompletely understood [10]. For instance dimer products may have lack of complementarity with the primers or show mismatches in the 3’ portion of the primers [11]. Improvements to avoid non-target amplification include the development of hot-start PCR, adding nucleotide tags in 5’ of both primers for Tag-driven PCR [11], heat-activatable primers [12] and cooperative primers [13].

In this study we show it is possible to use highly complementary primers in 5’ and avoid PD formation and develop efficient amplification assays by using a new asymmetric PCR method, COMplementary Primer ASymmetric PCR (COMPAS-PCR), using different forward and reverse primer concentrations. This counterintuitive approach will sequester, hence also protect, the limiting primer P^L^, while the excess primer P^X^ initiates linear amplification in presence of the target sequence. The reaction shifts towards exponential amplification when sufficient complementary amplicon strands are produced to serve as template material for P^L^. The PCR annealing temperature T_a_ is adjusted according to the lowest primer melt temperature T_m_ from P^X^, and the addition of short 3’ overhangs on the complementary primer improves the amplification efficiency. Asymmetric PCR, for which primer concentrations in a simplex reaction are unequal, is not a new concept and has been used for enrichment of one strand of the PCR amplicon with the purpose of improving probe detection. In particular, Linear-After-The-Exponential (LATE)-PCR improves such assay by compensating the decrease of melt temperature incurred to decreased concentrations of the limiting primer by extending it by a few nucleotides [14], further reaching optimum results when T_m_^L^–T_m_^X^ ≥ 5°C [15]. Opposite to LATE-PCR, COMPAS-PCR, by design, will begin by linear amplification before shifting to exponential amplification depending on sample target DNA concentration and P^X^ strand amplicon accumulation.

**Fig 1:**
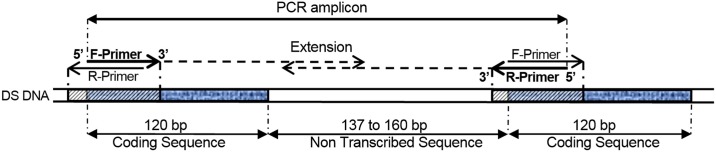
COMPAS-PCR using highly complementary primers applied to 5S rDNA direct repeat genes. Non Transcribed Sequence lengths are indicated according to sequence access numbers S73107 and LN835408-LN835422, varying from 137 and 138 bp for *S*. *salar* to 160 bp for *S*. *trutta*.

This COMPAS method was used to develop a 35 min three-primer duplex PCR for simultaneous identification of *S*. *salar*, *S*. *trutta* and hybrids using High Resolution Melt (HRM) analysis by targeting 5S rDNA, a multigene family organized in tandem direct repeat units [16]. Highly complementary primers were designed to have the same DNA target, and yet, structurally define an amplicon product due to the direct repeat structure of the target DNA (Fig 1 and Table 1). Specificity of the assay was enhanced by using 3’-penultimate point mutations in the reverse primers. Efficiency and specificity evaluation of variable elements such as degree of primer complementarity and concentration, T_a_, and melt temperatures for P^L^ and P^X^ are presented and discussed.

**Table 1:** Salmonid 5S rDNA primers for COMPAS-PCR.

Primer^a^	Specificity	Sequence^b^ 5’-3’	Tm^c^ °C	Prod. (bp)
5SNTS-23F		GCTTACGGCCATACCAGCCTGGG	64.4	
5SNTS-22R+2	*S*. *salar*	AGGCTGGTATGGCCGTAAGCGA	66.5	278^e^
5SNTS-23R+3	*S*. *salar*	AGGCTGGTATGGCCGTAAGCGAG	68.0	278^e^
5SNTS-23R+3-mamT	*S*. *salar*	AGGCTGGTATGGCCGTAAGCG**T**G	66.4	278^e^
5SNTS-23F-m1		GTTTACGGCCATACCAGCCTGGG	62.5	
5SNTS-23R+3-m2	*S*. *trutta*	AGGCTGGTATGGCCGTAAACGAT	65.2	300
5SNTS-23R+3-mamG-m2	*S*. *trutta*	AGGCTGGTATGGCCGTAAACG**G**T	64.2	300
5SNTS-23F-W1		G*Y*TTACGGCCATACCAGCCTGGG	62.4	
5SNTS-21R+57^d^	*S*. *trutta*	TGCATTTTGCAGCAGGAAGCT	62.7	225
5SNTS-23F-W1	*S*. *salar* & *S*. *trutta*	G*Y*TTACGGCCATACCAGCCTGGG	62.4	
5SNTS-22R+2-W1		AGGCTGGTATGGCCGTAA*R*CGA	63.2 & 64.7	278^e^ & 300

^a^The forward primers were used as the limiting primers and the reverse primers as the excess primers in the COMPAS-PCR 5S rDNA salmonid assay.

^b^Degenerate positions are italicized, penultimate mutations indicated in bold and *S*. *trutta* specific mutations are underlined.

^c^Tm were calculated using Oligo7 v7.60 [17], using a concentration of 50 and 600 nM for the limiting and excess primers respectively.

^d^Used in standard PCR.

^e^277 bp based on S73107.

## Materials and Methods

This study involved no endangered or protected species. Fish sampling was conducted according to the Norwegian Animal Research Authority (NARA) instructions and authorized by NARA approval ID 09/1723. Fish were sacrificed by a sharp blow to the head immediately after collection and all efforts were made to minimize suffering or distress. Salmonid sample information is shown in Table 2. The *S*. *salar* and *S*. *trutta* individuals used in this study for sequencing the 5S rDNA gene originated from 2 different areas in southern Norway: the Driva and the Lærdalselva river basins. Fins from fish individuals were clipped and preserved in 95% ethanol. DNA extraction was performed using Mole tissue kit on the Mole instrument (Mole Genetics, Lysaker, Norway; discontinued). Briefly, fin material was rinsed with distilled water and approximately 20 mg for each individual was transferred to a tube containing 100 μl Mole lysis buffer and 2 μl proteinase K from a 20 mg ml^-1^ stock solution (Merck, EC 3.4.21.14; www.merck.com). The samples were incubated at 65°C for 1 h and further processed using the Mole instrument according to the manufacturer’s instructions. The DNA concentration was measured using a NanoDrop ND-1000 spectrophotometer (www.nanodrop.com) and all samples were diluted to 2 ng μl^−1^ prior to performing qPCR.

**Table 2:** Sample origin and sequence information.

Isolate	Species	Country	Location	HRM analysed	Sequencing	Access number
S4825	*S*. *salar*	Norway	Driva River	Yes	ND	ND
S4826	*S*. *salar*	Norway	Driva River	Yes	5S rDNA	LN835408
S4827	*S*. *salar*	Norway	Driva River	Yes	5S rDNA	LN835409
S4829	*S*. *salar*	Norway	Driva River	Yes	5S rDNA	LN835410
L235	*S*. *salar*	Norway	Lærdalselva, Seltun	No	5S rDNA	LN835411
L236	*S*. *salar*	Norway	Lærdalselva, Seltun,	No	5S rDNA	LN835412
L237	*S*. *salar*	Norway	Lærdalselva, Skulehølen	No	5S rDNA	LN835413
L239	*S*. *salar*	Norway	Lærdalselva, Skulehølen	No	5S rDNA	LN835414
Moir-1	*S*. *salar*	Norway	Røssåga River	Yes	ND	ND
Moir-2	*S*. *salar*	Norway	Røssåga River	Yes	ND	ND
Moir-3	*S*. *salar*	Norway	Røssåga River	Yes	ND	ND
T4808	*S*. *trutta*	Norway	Driva River	Yes	5S rDNA	LN835415
T4816	*S*. *trutta*	Norway	Driva River	Yes	5S rDNA	LN835416
T4805	*S*. *trutta*	Norway	Driva River	Yes	5S rDNA	LN835417
T4821	*S*. *trutta*	Norway	Driva River	Yes	5S rDNA	LN835418
T4809	*S*. *trutta*	Norway	Driva River	No	5S rDNA	LN835419
T4815	*S*. *trutta*	Norway	Driva River	No	5S rDNA	LN835420
T107	*S*. *trutta*	Norway	Lærdalselva River	Yes	5S rDNA	LN835421
T124	*S*. *trutta*	Norway	Lærdalselva River	Yes	5S rDNA	LN835422
T132	*S*. *trutta*	Norway	Lærdalselva River	Yes	ND	ND
ST4818	*S*. *salar* X *S*. *trutta*	Norway	Driva River	Yes	ND	ND
ST4819	*S*. *salar* X *S*. *trutta*	Norway	Driva River	Yes	ND	ND
ST4810	*S*. *salar* X *S*. *trutta*	Norway	Driva River	Yes	ND	ND
ST4813	*S*. *salar* X *S*. *trutta*	Norway	Driva River	Yes	ND	ND
ST4817	*S*. *salar* X *S*. *trutta*	Norway	Driva River	Yes	ND	ND
ST4811	*S*. *salar* X *S*. *trutta*	Norway	Driva River	Yes	ND	ND
ST4812	*S*. *salar* X *S*. *trutta*	Norway	Driva River	Yes	ND	ND
234	*O*. *tshawytscha*	U.S.A.	Oregon	Yes	ND	ND
252	*O*. *tshawytscha*	U.S.A.	Oregon	Yes	ND	ND

All primers designed for this study are shown in Table 1 and primer names include length, overhang position into the NTS as well as presence, if any, of penultimate destabilizing point mutations, degenerate nucleotide positions and *S*. *trutta* specific mutations.

**Fig 2:**
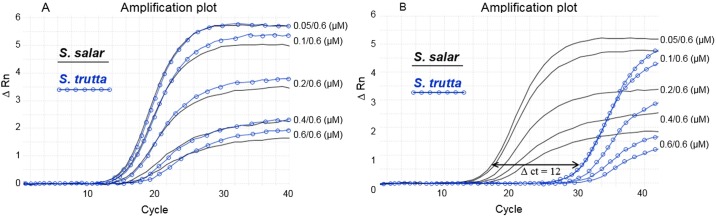
COMPAS-PCR principles. Asymmetric concentration effect for 5S rDNA qPCR highly complementary primers tested on *S*. *salar* L235 and *S*. *trutta* T107. Concentration of the forward primer 5SNTS-23F in A and B ranges from 0.6 to 0.05 μM while a constant concentration of 0.6 μM was used for the reverse primer 5SNTS-22R+2 in A and 5SNTS-23R+3 in B.

Results shown in Fig 2 were produced with an ABI 7500 qPCR machine (Life Technologies, Applied Biosystems) using Mesa Blue master mix (Eurogentec). The final PCR reaction volume of 25 μl contained 12.5 μl mastermix, 0.6 μM of either 5SNTS-23R+2 or 5SNTS-23R+3 reverse primer and 5SNTS-23F forward primer at concentrations varying from 0.6 to 0.05 μM, 2.5 μl sample (5 ng DNA), completed with sterile deionized water. Cycling conditions were as follows: 5 min denaturing step at 95°C, followed by 40 cycles at 95°C for 20 s, 62°C for 30 s and 72°C for 60 s.

For sequencing, PCR amplifications were carried out with a CFX96 BioRad thermocycler (Bio-Rad, Hercules, CA, USA) under the following conditions: a denaturing step for 30 s with iProof (BioRad) at 98°C, followed by 40 cycles of 98°C for 10 s, 65°C for 10 s and 72°C for 15 s.

Cycle sequencing was performed in both directions using amplification primers and BigDye Terminator v3.1 kit (Life Technologies, Applied Biosystems). PCR template was diluted 10 fold in ddH2O and 1 μl was used with 0.5 μl Terminator mix, 0.32 μl 10 μM forward or reverse primer, 1.75 μl Terminator 5X buffer in a final volume of 10 μl. Cycle sequencing was performed using an ABI 7500 qPCR machine as following: 96°C for 1 min followed by 28 cycles of 96°C for 10 s, 50°C for 5 s and 60°C for 4 min. Sequence purification was performed using BigDye XTerminator Purification kit (Life Technologies, Applied Biosystems) adding 10 μl XTermination solution and 45 μl Sam solution to each PCR sample well, final volume of 65 μl. The PCR plate was then sealed and vortexed for 30 min prior to being processed by an ABI3730XL DNA analyzer (Life Technologies, Applied Biosystems). Trace files analyses were performed using CodonCode Aligner v5.1.5 (CodonCode Corporation), sequence alignments were performed using MultAlin [18] and edited for presentation using GeneDoc v2.7 software. The two three primer duplex COMPAS-PCR for HRM identification of *S*. *salar*, *S*. *trutta* and hybrids were carried out under the following conditions: a denaturing step for 2 min (SsoFast EvaGreen) at 98°C, followed by 30 cycles of 98°C for 1 s and 70°C for 1 s. Primers 5SNTS-23F-W1 100 nM and 5SNTS-23R+3-mamT 300 nM were combined with either 5SNTS-23R+3-mamG-m2 500 nM or 5SNTS-21R+57 2000 nM. Melt curve analysis was performed from 69°C to 92°C using 0.5°C increments and HRM analysis was performed using Precision Melt analysis software (Bio-Rad) by setting the difference curve analysis between 78 and 82°C. Oligo7 v7.60 [17] was used for calculating Tm and predicting dimer and hairpin formation for primers. PCR products were electrophoresed on a 1.4% agarose gel (Agarose I biotechnology grade, VWR) in 2 x TAE buffer (VWR), with a 100 bp ladder (NEB) and visualized with GelRed (Biotium; www.biosciences.co.uk) staining.

## Results and Discussion

### COMPAS-PCR

Choice of target DNA for designing PCR assays will depend on the purpose of the application. Taxonomic group identification or genetic diseases diagnostic will often have distinct requirements, in particular to achieve specificity and required sensitivity. High copy number target genes are useful as they help improve sensitivity of the assay although they may lack sufficient sequence variability to insure specificity. This shortcoming may be mitigated when a non-coding sequence is associated in the immediate proximity of the targeted conserved transcripted target DNA gene. This is the case with rDNA genes present in multiple copy number which display conserved sequences homogenized though concerted evolution [19]. As expected, the degree of sequence variability is highest for the non-coding sections such as the non-transcribed spacer (NTS) of 5S rDNA [20]. Hence, several methods have been developed targeting 5S rDNA for molecular diagnostic of parasites [21, 22], squids [23], and fish [24–31]. The genetic organization of direct repeat multi-copy gene families gives the theoretical possibility of designing a pair of primers targeting the same locus and yet amplifying a distinct product as shown in Fig 1. However, such a design would transgress the conventional practice of avoiding primer complementarity when developing PCR assays [32, 33]. In this study we show that COMPAS-PCR enables such a design to be successfully used for the development of efficient PCR assays primarily by using asymmetric primer concentrations. The choice of short length repeat unit targets, such as the 5S rDNA genes, further enables the amplification of short amplicons e.g. around 200bp, which are better suited when analyzing possibly degraded samples [34].

DNA based diagnosis for salmonids, and in particular identification of the closely related *S*. *salar* and *S*. *trutta* species, has previously been performed by 5S rDNA “universal” PCR targeting the conserved 120bp Coding DNA Section (CDS). The corresponding amplification products comprise the length variable NTS, used in the method for species identification by gel analysis [24], as well as for *S*. *salar* x *S*. *trutta* hybrid identification [25]. Simplex PCR species specific amplification was later developed by targeting the NTS section of the 5S rRNA gene, albeit still requiring gel analysis [31]. Other methods have been developed for salmonid identification such as PCR-RFLP applied to the p53 and mitochondrial tRNAGlu/cytochrome b genes [35, 36] or COI gene very short amplicon simplex PCR [37] and COI multiplex probe qPCR [38]. However, most of these methods require time consuming post PCR product analysis and none of them include *S*. *trutta* identification. Hence, COMPAS-PCR principles were initially developed to provide a user-friendly rapid single tube qPCR assay for unambiguous identification of *S*. *salar*, *S*. *trutta* and hybrids without the need to run post amplification analysis. The primers were designed at the start of the 120 bp CDS of the 5S rDNA with a short part extending over the non-transcribed sequence (NTS) section. This design over the CDS/NTS intersection aimed at strengthening the robustness of the assay by increasing primer stability over the conserved CDS section, to avoid false negatives, and seek for specificity over the NTS section. The forward primer starts at position 1 of the CDS extending into the CDS, and the reverse primers initiate in the start region of the CDS extending into the NTS section with up to 3 nucleotides overhang in 3’. The organization of the target in tandem direct repeats insures that at least 1 unit of each section may be amplified. Hence, depending on how many direct repeats are present, several products consisting of 2 or more sections may also be amplified as previously reported using standard PCR [25]. The COMPAS-PCR primers purposely overlap the same target section and are highly complementary to each other (Fig 1). This primers’ self-complementarity is expected to compete with target priming, hence strongly inhibit target amplification, the reason for which this configuration is avoided when designing PCR assays [32, 33].

In order to favor priming to target DNA, primers’ self-priming was unlocked by using asymmetric primer concentrations by decreasing either the forward or the reverse primer concentration until optimal PCR amplification was reached. As shown in Fig 2A, a strong and equal amplification was progressively generated for both *S*. *salar* and *S*. *trutta* as the forward primer concentration was decreased. Asymmetric PCR has been previously described for enhancing probe based detection during which the PCR shifts from exponential to linear amplification to favor probe hybridization to its target single stranded sequence using LATE-PCR [14, 39]. With COMPAS-PCR the primers are highly complementary and the asymmetric PCR has an opposite pattern shifting from linear to exponential amplification, effectively alleviating the target amplification inhibition otherwise observed with complementary primers. During the first amplification cycles the concentration-limited primer P^L^ will be mainly sequestered by the excess primer P^X^ such that mostly P^X^ initiated linear amplification will take place. As linear products complementary to P^L^ accumulate while sequestering P^X^ complementary primer concentration decreases, P^L^ target priming and subsequent amplification will be favored.

Forward and reverse primers in Fig 2 have an overlapping complementary perfect match of 20 nucleotides. Primers were designed based on S73107 sequence information. Testing of reverse primer overhang extension into the NTS section showed that position +3 gave a sharp differentiation between *S*. *salar* and *S*. *trutta* (Fig 2B). The reverse primer in Fig 2B has an additional nucleotide in 3’ compared with the reverse primer used in Fig 2A. Sequencing showed the presence of a point mutation at the corresponding 3’ reverse primer terminal position differentiating *S*. *salar* from *S*. *trutta* in Fig 2B. Amplification products were sequenced in order to identify species specific variations and use them for further developing a one-tube assay for simultaneous identification of *S*. *salar*, *S*. *trutta* and hybrids as detailed in the next sections.

### Primer Dimers

**Fig 3:**
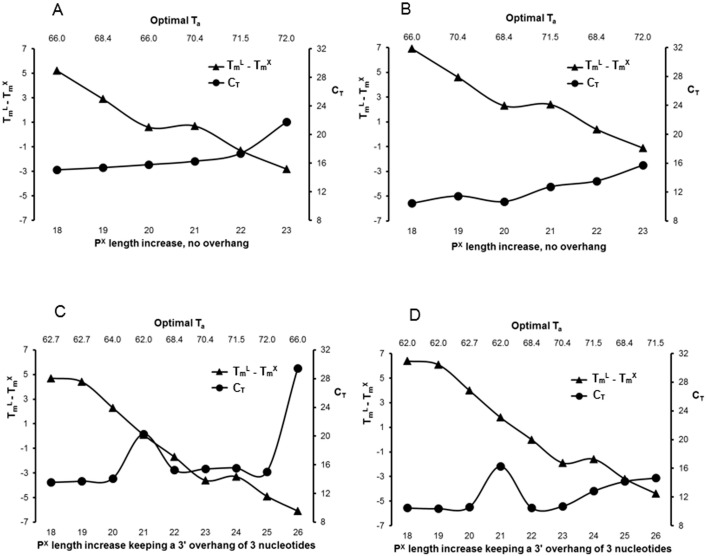
COMPAS-PCR optimization. Effect of length variations in the reverse excess primer (P^X^) with a 23 bp complementary forward limiting primer (P^L^) using 5 ng genomic *S*. *salar* DNA and SsoFast Evagreen mastermix. P^L^ = 5NTS-23F at a concentration of 50 (A & C) or 200 nM (B & D) and P^X^ at 600 nM (A, B, C & D). In each experiment P^X^ 3’ end is unchanged, either with no overhang (A & B) or with a 3 nucleotide overhang (C & D) after the forward P^L^ 5’ complementary end. P^X^ length is incremented in 5’ from 18 nucleotides until it reaches the 3’ end of P^L^ at 23 nucleotides resulting in a 100% complementarity between P^X^ and P^L^ (A & B), or 26 nucleotides (C & D). A gradient PCR was run varying the annealing temperature (T_a_) from 62 to 72°C. The P^X^ nucleotide length increase is shown on the lower horizontal axis. The difference between melt temperature of the limiting primer (T_m_^L^) and melt temperature of the excess primer (T_m_^X^) is reported on the left-hand vertical axis as T_m_^L^—T_m_^X^ and plotted as triangles. For each P^X^, the corresponding optimal T_a_ is reported on the upper horizontal axis. The resulting Cycle Threshold C_T_ is reported on the right-hand vertical axis and plotted as circles.

Primer dimer formation is typically described as resulting from the association in 3’ of 2 primers during a PCR reaction, followed with DNA extension resulting in the production of a non-target amplicon shorter than the sum of the length of the two primers. Sequencing of such primer dimers have shown that primer mismatches in 3’ may be present and that unknown short nucleotide sequences may also be present in its center [10, 11] showing a more complex situation than first thought. A possible explanation for incorporation of non-primer sequences in primer dimers may be the involvement of genomic DNA in the non-target annealing of the primers close to each other [40]. COMPAS-PCR will effectively reduce the development of “genomic” primer dimers as the limiting primer is sequestered by the excess primer dimer during the linear phase. After target sequence has accumulated and excess primer concentration has decreased sufficiently, the limiting primer will be released resulting in exponential target amplification. When applied to tandem direct repeat targets such as the 5S rDNA used in this study, primer complementarity and extension in 3’ would theoretically not hinder complementarity to the target as the extended primers would still find a perfect match on the target sequence. However, the resulting 100% self-primer match produces a less efficient amplification (Fig 3A and 3B).

### Effect of Primer Length and Complementarity Positioning

Primer length and degree of complementarity was tested by using the forward 23 bp P^L^ 5NTS-23F paired with a complementary reverse P^X^ varying in length from 18 bp up to 26 bp. Two groups of reverse P^X^ were tested forming either a 3 nucleotide overhang or a blunt end in 3’ when paired with the forward P^L^. The length of the reverse P^X^ was increased in 5’ from 18 bp length to reach either 26 bp with the 3 nucleotide 3’ overhang group or 23 bp for the 3’ blunt end therefore forming 100% complementarity when paired to the forward P^L^. Two concentrations, 50 and 200 nM, were tested for P^L^ whereas P^X^ always had a concentration of 600 nM. Melting temperatures for both P^L^ and P^X^ were calculated taking into account primer length, composition and concentration, and displayed as Tm^L^–Tm^X^ in Fig 3. Finally, a gradient qPCR was performed, amplification specificity was checked by melt curve analysis and the resulting optimal annealing temperatures as well as cycle threshold values were reported in Fig 3 and plotted together with Tm^L^–Tm^X^ and P^X^ length. As expected, the melt temperature difference between P^L^ and P^X^ decreases as P^X^ length increases and becomes negative before P^X^ length matches P^L^ length at 23bp (as expected since P^X^ has a higher concentration than P^L^). Best C_T_ values are obtained with the highest Tm^L^–Tm^X^ values, up to + 6.9°C difference (Fig 3B), and remain similar when P^X^ is increased by up to two nucleotides. This best C_T_ value corresponds to the smallest tested P^X^ at 18bp. In general, C_T_ values increased moderately and regularly as P^X^ length increased, showing reduced PCR efficiency, except for reverse P^X^ primer 5SNTS-21R+3 (Fig 3C and 3D) characterized by a C_T_ value increased by approximately 5 cycles. This exception was consistently observed whether using a concentration of 50 or 200 nM for the forward P^L^ primer. As both reverse primers with either one less nucleotide or one more, 20 and 22 nucleotides in length respectively, fitted the general trend, the reduced efficiency for the 21 nucleotide reverse primer must be associated to its 5’ end nucleotide. Oligo7 showed that no hairpin structure was present and that dimer formations were similar to those found for 5SNTS-20R+3 and 5SNTS-22R+3. Terminal dangling ends in 5’ [41], with 5SNTS-21R+3 either associated to the forward primer or to the target DNA, could possibly partly explain this increased C_T_ value. However, primer 5SNTS-18R which has the same 5’ terminal end, showed no reduced efficiency, indicating that the 3’ overhang present with 5SNTS-21R+3 seems also involved when associated to the 5’ specific terminal position for reducing the efficiency of the PCR.

As expected, optimal annealing temperature is found to generally increase as the tested P^X^ is extended. Moreover, and in particular for P^X^ without any overhang, robustness of the assay, as defined by the range of suitable T_a_ for the assay, decreases as P^X^ increases in size (data not shown). When an overhang is present, limits of the assay seem to have been reached at 26 bp when P^L^ concentration is 50 nM (Fig 3C).

Testing the 100% complementary primer pair 5NTS-23F / 5NTS-23R required using asymmetric primer concentrations to produce target results whereas the presence of a short overhang of one to three nucleotides on at least one side enabled target amplification without requiring asymmetric concentrations when using SsoFast EvaGreen kit. However, important variations were observed when comparing PCR mastermix kits and assays using 100% complementary primers had low sensitivity. PCR efficiency was evaluated by establishing standard curves using tenfold serial dilutions of *S*. *salar* genomic DNA in duplicates. A dynamic range over 5 logs was obtained starting from 5ng template DNA producing a PCR efficiency and correlation values of 97.0%– 101.8% and 0.993 respectively with primers 5NTS-23F at 50 or 200 nM and 5NTS-18R+3 at 600 nM.

### Sequencing

**Fig 4:**
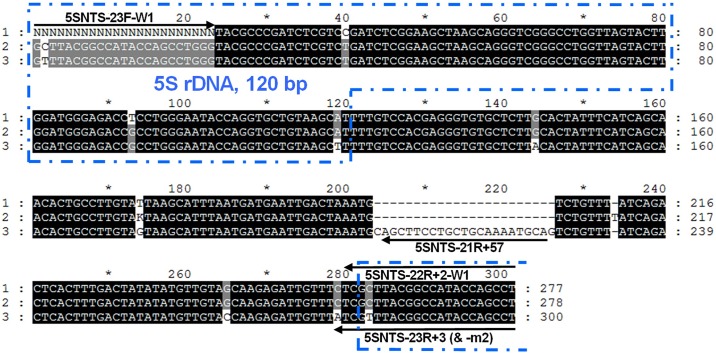
5S rDNA sequence alignments with selected primers. Coding DNA sequence is indicated by the dotted boxing. Non-sequenced positions are shown (N) and gaps (-) are inserted for alignment purposes. Product length is indicated at the end of each sequence while sequence numbering along the sequences is performed according to the alignment. 1, *Salmo salar* S73107; 2, *Salmo salar* LN835408 to LN835414, all 7 sequences are identical; 3, *Salmo trutta* LN835408 to LN835414, all 8 sequences are identical. Positioning of the primers is shown on the figure and have the following specificity: “universal” forward primer 5SNTS-23F-W1 + “universal” reverse primer 5SNTS-22R+2-W1 for amplifying both *S*. *salar*, *S*. *trutta* and *S*. *salar* X *S*. *trutta* hybrids; forward primer 5SNTS-23F-W1 & Reverse primer 5SNTS-23R+3-mamT for amplifying *S*. *salar*, and *S*. *salar* X *S*. *trutta* hybrids; “universal” forward primer 5SNTS-23F-W1 & Reverse primer 5SNTS-23R+3-mamG-m2 for amplifying *S*. *trutta*, and *S*. *salar* X *S*. *trutta* hybrids; “universal” forward primer 5SNTS-23F-W1 & Reverse primer 5SNTS-21R+57 for amplifying specifically *S*. *trutta*, and *S*. *salar* X *S*. *trutta* hybrids.

The *S*. *salar* and *S*. *trutta* 5S rDNA sequences from this study, accession numbers LN835408 to LN835422 shown in Table 2, are available at the European Nucleotide Archive. The primers used for COMPAS-PCR were initially designed based on the published *S*. *salar* 5S rDNA sequence S73107 [24]. The primers 5SNTS-23F and 5SNTS-22R+2 were used for amplifying 5S rDNA from *S*. *salar* and *S*. *trutta* by COMPAS-PCR using asymmetric concentrations (Fig 2), and for sequencing. The primary objective of sequencing the produced amplicons was to identify the 3’ terminal SNP deduced from the *S*. *salar* specificity shown by 5SNTS-23R+3 compared with 5SNTS-22R+2 (Fig 2). All 8 *S*. *trutta* produced identical sequences confirming the presence of nucleotide A instead of C in position 279 (Fig 4) and showed an insertion of 23 bp in the NTS at position 204. Additionally, the *S*. *trutta* samples showed the presence of an ambiguity in position 2 of the 120 bp coding DNA sequence (CDS). Both nucleotides, C, also present in S73107, and T were found in this position. This ambiguity was consistently observed in this position for both repeated CDS parts present in the amplicon. New forward and reverse sequencing primers, respectively 5SNTS-23F-m1 and 5SNTS-23R+3-m2, using T instead of C for CDS position 2 unambiguously showed the presence of a T for all 8 sequenced *S*. *trutta*. Moreover, 4 additional SNPs were observed in all *S*. *trutta* sequences compared with the *S*. *salar* sequences (Fig 4 and Figs A-H in S1 File).

All 7 *S*. *salar* individuals produced identical sequences which showed 4 discrepancies compared with S73107 (Fig 4 and Figs A-H in S1 File) including an insertion in the NTS (position 234 in Fig 4) as well as an intra-individual variation at position 174 in the NTS (Fig 4 and Figs E and F in S1 File). Nucleotides T and G were identified at position 174, corresponding to the nucleotides reported in *S*. *salar* S73107 and *S*. *trutta* (this study) respectively. Direct sequencing from PCR products used in the present study takes advantage of the electropherograms quantitative properties which show the presence of different nucleotides at a given position when analyzing PCR products from multi-copy target DNA. In particular this has been exploited in the field of epigenetics with the development of direct bisulfite PCR sequencing [42, 43]. Direct PCR product sequencing can also be used to uncover heteroplasmy, an intra-individual multi-copy gene variation found for example in plastids such as mitochondria, for which heteroplasmy may have been under-reported [44]. Although not reported in S73107 5S rDNA sequence, intra-individual variation may have gone unnoticed as only 2 clones were sequenced for determining this sequence [24]. This intra-individual variation was identified in all 7 *S*. *salar* individuals originating from 2 different Norwegian river basins and therefore shows stability within the species in this geographic area. Analyses of individuals from new localities would be necessary to evaluate the extent of the presence of this intra-individual variation across the species.

### One-Tube *S*. *salar* and *S*. *trutta* Specific Assay

The additional sequence information showing differences between the 2 species was exploited for designing and testing additional species specific primers for developing a single tube COMPAS-qPCR identification assay for *S*. *salar*, *S*. *trutta* and their hybrids (Table 1).

**Fig 5:**
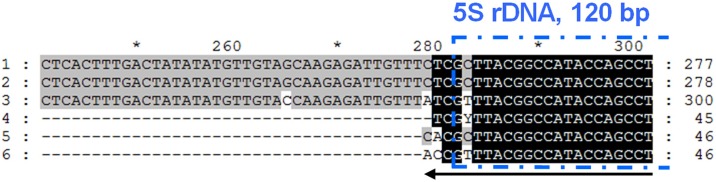
Salmo COMPAS-PCR reverse primer specificity including penultimate mismatch design. Reverse complement of the primers are shown in the alignment, the 5S rDNA coding sequence is indicated by the dotted box. 1, *Salmo salar* S73107; 2, *Salmo salar* LN835408 to LN835414, all 7 sequences are identical; 3, *Salmo trutta* LN835408 to LN835414, all 8 sequences are identical; 4, Reverse primer 5SNTS-22R+2-W1 for amplifying both *S*. *salar*, *S*. *trutta* and *S*. *salar* X *S*. *trutta* hybrids; 5, Reverse primer 5SNTS-23R+3-mamT for amplifying specifically *S*. *salar*, and *S*. *salar* X *S*. *trutta* hybrids; 6, Reverse primer 5SNTS-23R+3-mamG-m2 for amplifying specifically *S*. *trutta*, and *S*. *salar* X *S*. *trutta* hybrids.

In particular the 23 bp *S*. *trutta* specific insert in position 204–226 and the SNP in position 279 (Fig 4) were exploited for developing two separate *S*. *trutta* specific assays. A single common forward primer was used for both species using the first 23 bp of the CDS. To take into account the SNP identified in position 2 (and 283 in the following CDS), both A & G nucleotide found in this position were included in the degenerate “universal” primer 5SNTS-23F-W1. However, a mismatch would have had little effect since the SNP is positioned at the 5’ end of the forward primer. Conversely, the same SNP is positioned at the 3’ end of the reverse COMPAS primers where a mismatch will have a higher impact with increased miss priming effect. This SNP was used together with SNP position 279 placed at the 3’ terminal end of reverse primer 5SNTS-23R+3-m2 for increased *S*. *trutta* specificity. Assay specificity robustness may further be improved by strategies using penultimate mutations to strengthen the effect of a primer 3’ terminal specific mutation [2, 6] and was used for enhancing this assay. All three mutation possibilities were tested for both species showing T best for *S*. *salar* specific assay implemented in 5SNTS-23R+3-mamT reverse primer and G best for *S*. *trutta* specific assay implemented in 5SNTS-23R+3-mamG-m2 reverse primer (Table 1 and Fig 5). Finally, a second *S*. *trutta* specific assay was developed based on the specific 23 bp insert using primer 5SNTS-21R+57 amplifying a 225 bp product when used with 5SNTS-23F-W1. Discrimination between *S*. *salar* and *S*. *trutta* was achieved with all 3 assays while hybrids were also detected as expected in all 3 assays, each assay producing a size specific amplicon (Fig A in S2 File).

The species specific reverse primers of the simplex assays were combined with the same forward primer in order to develop a one-tube assay for discrimination of both *S*. *salar*, *S*. *trutta* and hybrids. The “universal” forward primer was associated with the penultimate mutation specific *S*. *salar* reverse primer and the penultimate mutation specific *S*. *trutta* reverse primer in a three primer duplex COMPAS-PCR assay. Finally, a second three primer duplex assay was developed by replacing the penultimate mutation specific *S*. *trutta* reverse primer with the *S*. *trutta* insert specific primer. The same assay stringency conditions used for the COMPAS-PCR salmo simplex using a 2-step PCR, 1 s annealing and 1 s amplification, was successfully applied to the duplex assays achieving amplification and melt analysis within 35 min. Both assays showed species specific products as well as the presence of both products for hybrid individuals when run on a gel (Fig B in S2 File).

**Fig 6:**
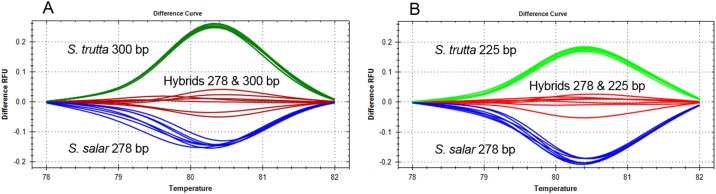
High Resolution Melt analysis of two three-primer duplex COMPAS-PCR for the differentiation of *S*. *salar*, *S*. *trutta* and hybrids. Primers 5SNTS-23F-W1 100 nM and 5SNTS-23R+3-mamT 300 nM were combined with either 5SNTS-23R+3-mamG-m2 500 nM (A) or 5SNTS-21R+57 2000 nM (B). Color code: In both (A) and (B) the lower blue cluster shows all analyzed *S*. *salar* with a 278bp PCR product. The top green cluster shows all analyzed *S*. *trutta*, with a specific PCR product in A and B, respectively 300bp and 225bp. The central red cluster shows all analyzed hybrids with mixed PCR products, 278bp + 300bp in A and 278bp + 225bp in B. The hybrid cluster has been chosen as the reference cluster in both (A) and (B).

In order to avoid the necessity to run a gel, a High Resolution Melt Analysis was successfully implemented for discrimination of the 3 groups (Fig 6). The specificity and sensitivity of these two assays will depend on the conservation of the DNA variations used to characterize and identify each species. The fish individuals used in this study for sequencing the 5S rDNA gene originated from 2 different river basins in southern Norway. Hence, additional testing is required to assess the validity and efficiency of these assays for target individuals originating from other geographic areas. Non-target amplification, other than differentiating between the three targets, was assessed by testing two *Oncorhynchus tshawytscha* individuals (Table 2), a salmonid species belonging to the sub-family Salmoninae along with *S*. *salar* and *S*. *trutta*. DNA quality of the *O*. *tshawytscha* individuals had been previously successfully tested [45] and gave negative results when used with the *S*. *salar* and *S*. *trutta* specific assays (Fig B in S2 File). However, additional testing with other species would be required to validate these assays.

## Concluding Remarks

The COMPAS-PCR method was demonstrated for, but is not restricted to the identification of fish species. Using almost fully complementary primers targeting the same sequence may apply to any tandem direct repeats DNA motifs of interest as target sequences. Ribosomal genes and in particular the 5S r-DNA tandem direct repeats are found in all eukaryotic cells [46, 47] and are therefore suitable for developing specific complementary-primer assays for other taxon and species than salmonids. DNA repeat sequences are common in Eukaryote genomes and reported composing more than 50% of the human genome [47]. Hence, the general COMPAS-PCR principles will help develop new DNA amplification strategies taking advantage of these repeated DNA structures.

## Supporting Information

S1 File Sequencing electropherograms showing sequence variations, both single nucleotide polymorphisms (SNPs) and indels, between *S*. *salar* and *S*. *trutta*. 4 individuals are shown for each species: S4829, S4826, L235 and L237 for *S*. *salar* and T4809, T4815, T107 and T124 for *S*. *trutta*. Primers 5SNTS-23F and 5SNTS-23R+3 are used for *S*. *salar* and primers 5SNTS-23F-m1 and 5SNTS-23R+3-m2 are used for *S*. *trutta*. Coding DNA sequence is indicated by the dotted boxing.
(PDF)

S2 File Gel runs of simplex and duplex COMPAS-PCR for the identification of *S*. *salar*, *S*. *trutta* and hybrids. The closely related species *O*. *tshawytscha* was included to challenge specificity, showing no amplification.
(PDF)
