# Supplementary material for: COMplementary Primer ASymmetric PCR (COMPAS-PCR) Applied to the Identification of Salmo salar, Salmo trutta and Their Hybrids
Source: PLoS One. 2016 Oct 26;11(10):e0165468. doi: 10.1371/journal.pone.0165468 (PMC5082663; doi:10.1371/journal.pone.0165468)
Supplement: S1 File — 4 individuals are shown for each species: S4829, S4826, L235 and L237 for S. salar and T4809, T4815, T107 and T124 for S. trutta. Primers 5SNTS-23F and 5SNTS-23R+3 are used for S. salar and primers 5SNTS-23F-m1 and 5SNTS-23R+3-m2 are used for S. trutta. Coding DNA sequence is indicated by the dotted boxing. (PDF) [file pone.0165468.s001.pdf]

S1 File. Sequencing electropherograms showing sequence variations, both single nucleotide polymorphisms (SNPs) and indels, between *S. salar* and *S. trutta*. 4 individuals are shown for each species: S4829, S4826, L235 and L237 for *S. salar* and T4809, T4815, T107 and T124 for *S. trutta*. Primers 5SNTS-23F and 5SNTS-23R+3 are used for *S. salar* and primers 5SNTS-23F-m1 and 5SNTS-23R+3-m2 are used for *S. trutta*. Coding DNA sequence is indicated by the dotted boxing.

A

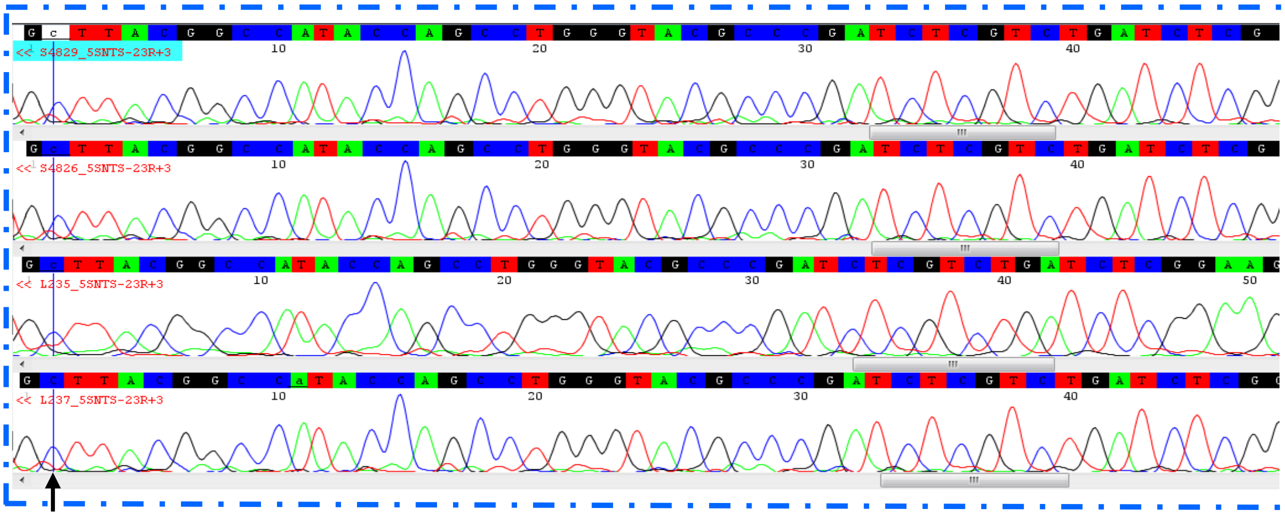

B

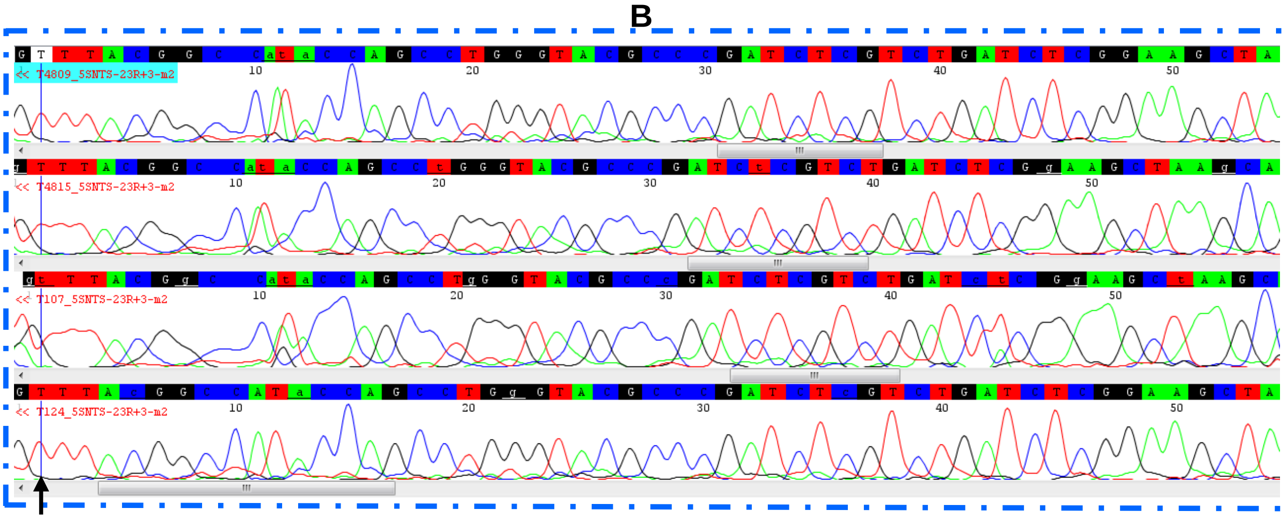

Figures A and B. Nucleotide number 2 marked by an arrow is cytosine for *S. salar* (A) and thymine for *S. trutta* (B).

C

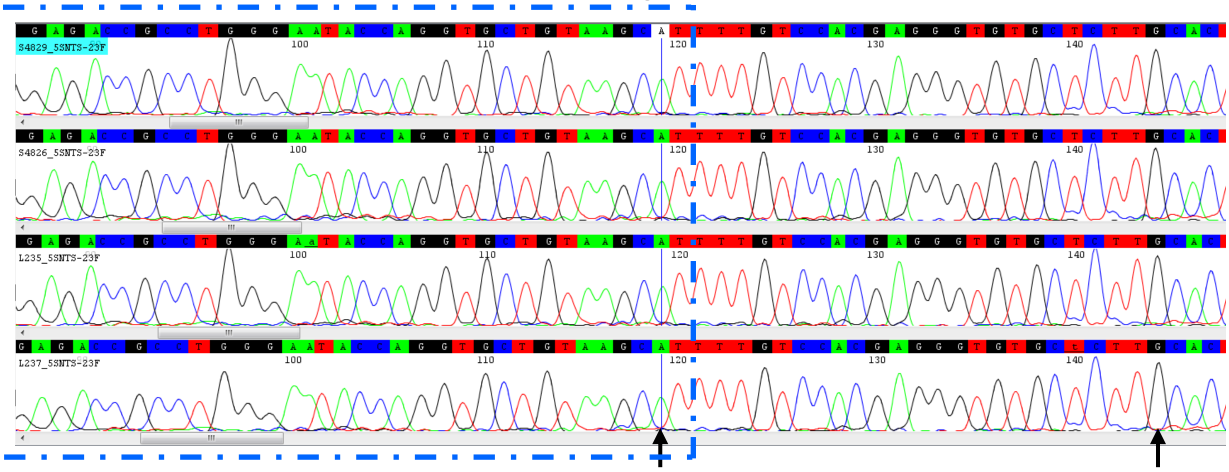

D

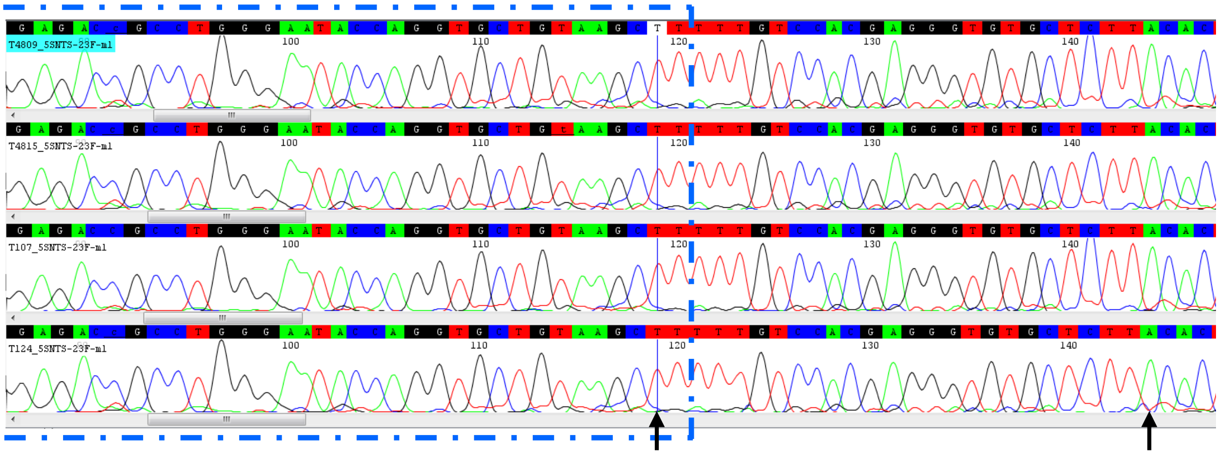

Figures C and D. Nucleotides number 119 and 144 marked by an arrow are respectively adenine and guanine for *S. salar* (A) and thymine and adenine for *S. trutta* (B).

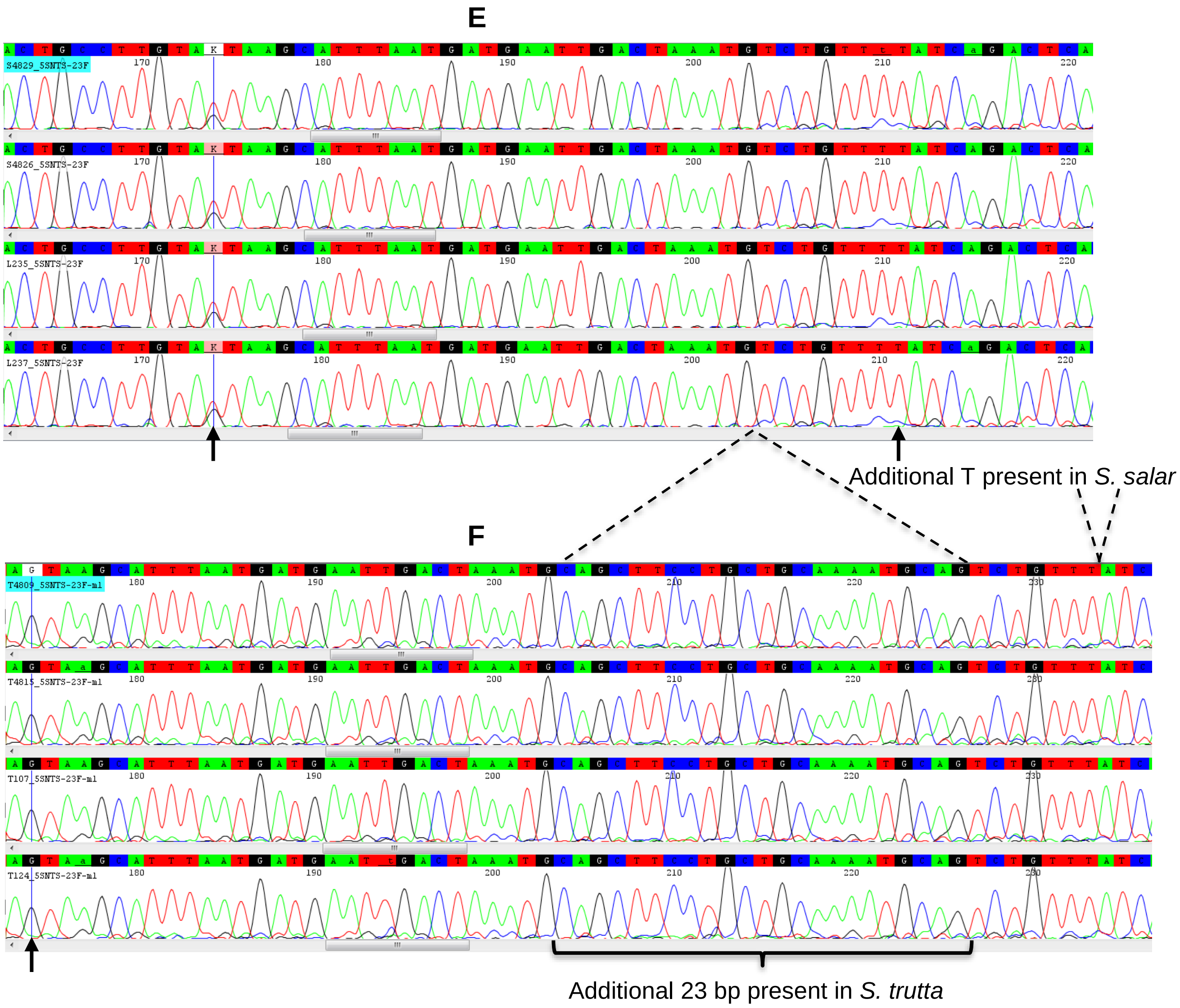

Figures E and F. Nucleotide number 174 marked by an arrow is thymine and guanine (heteroplasmy) for *S. salar* (A) and guanine for *S. trutta* (B). *S. trutta* has an additional 23 bp sequence starting at position 204 (B). A single nucleotide insertion, thymine, was present in position 211 (234 in Fig 4) of *S. salar* (A).

G

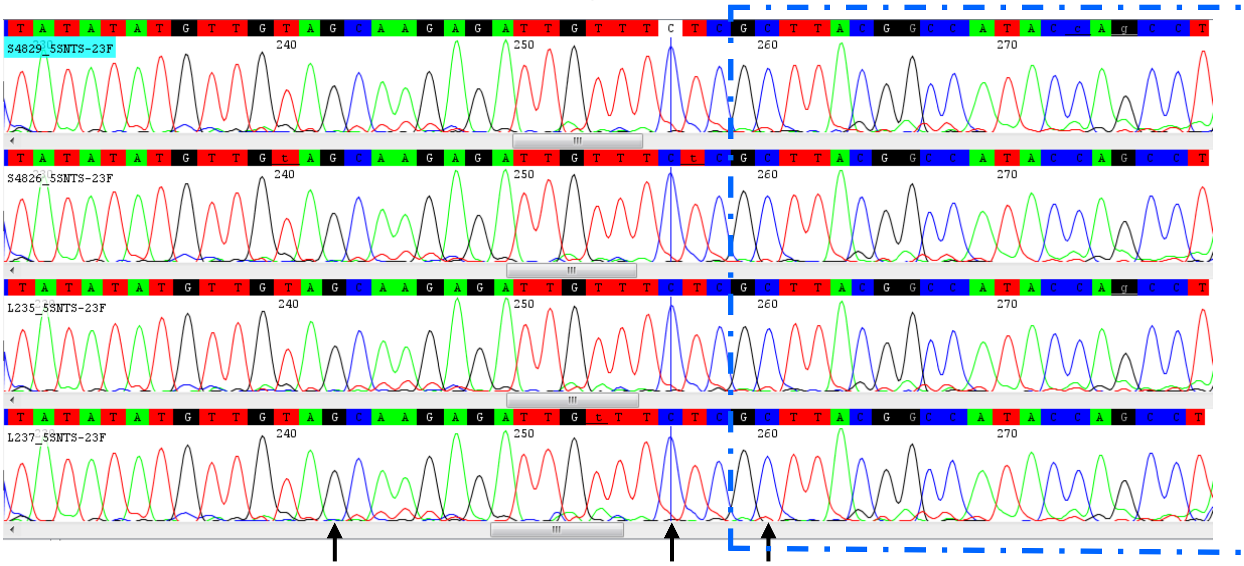

H

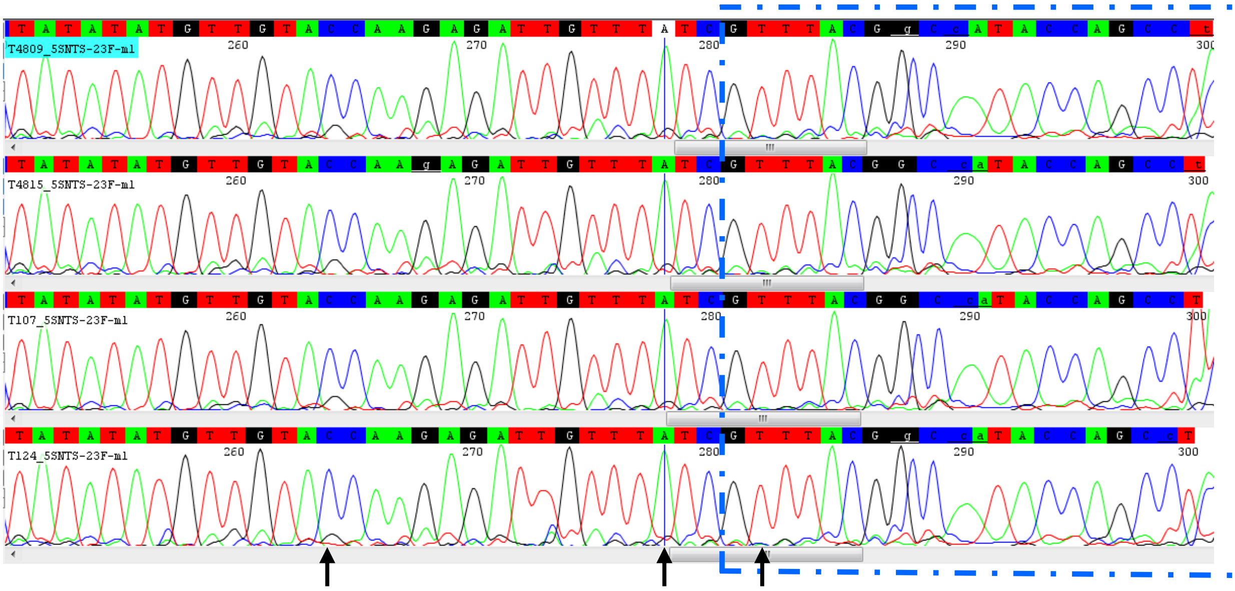

Figures G and H. For *S. salar* (A), nucleotides at positions 242 (265 in Fig 4), 256 (279 in Fig 4) and 260 (283 in Fig 4) marked by an arrow are respectively guanine, cytosine and cytosine. The corresponding positions for S. trutta (B) at 264 (265 in Fig 4), 278 (279 in Fig 4) and 282 (283 in Fig 4) marked by an arrow are respectively cytosine, adenine and thymine.
