# Supplementary material for: COMplementary Primer ASymmetric PCR (COMPAS-PCR) Applied to the Identification of Salmo salar, Salmo trutta and Their Hybrids
Source: PLoS One. 2016 Oct 26;11(10):e0165468. doi: 10.1371/journal.pone.0165468 (PMC5082663; doi:10.1371/journal.pone.0165468)
Supplement: S2 File — The closely related species O. tshawytscha was included to challenge specificity, showing no amplification. (PDF) [file pone.0165468.s002.pdf]

S2 File. Gel runs of simplex and duplex COMPAS-PCR for the identification of *S. salar*, *S. trutta* and hybrids. The closely related species *O. tshawytscha* was included to challenge specificity, showing no amplification.

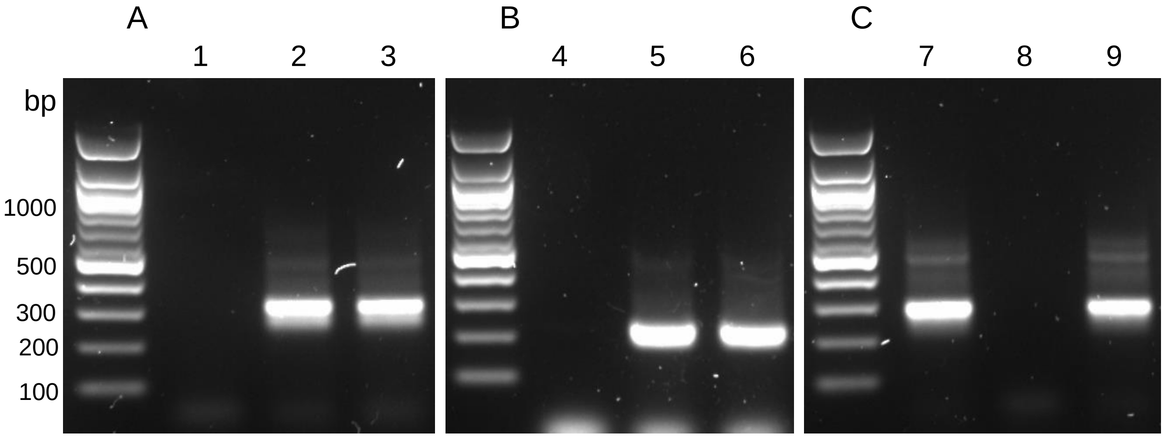

Figures A, B and C. Simplex COMPAS-PCR for *S. trutta* and hybrids (A & B) and *S. salar* and hybrids (C) using primers 5SNTS-23F-W1 + 5SNTS-23R+3-mamG-m2 (300bp, A); 5SNTS-23F-W1 + 5SNTS-21R+57 (225bp, B); and 5SNTS-23F-W1 + 5SNTS-23R+3-mamT (278bp, C). Lanes 1, 4 & 7: *S. salar* S4826; lanes 2, 5 & 8: *S. trutta* T4816; and lanes 3, 6 and 9: *S. salar* x *S. trutta* hybrid ST4819.

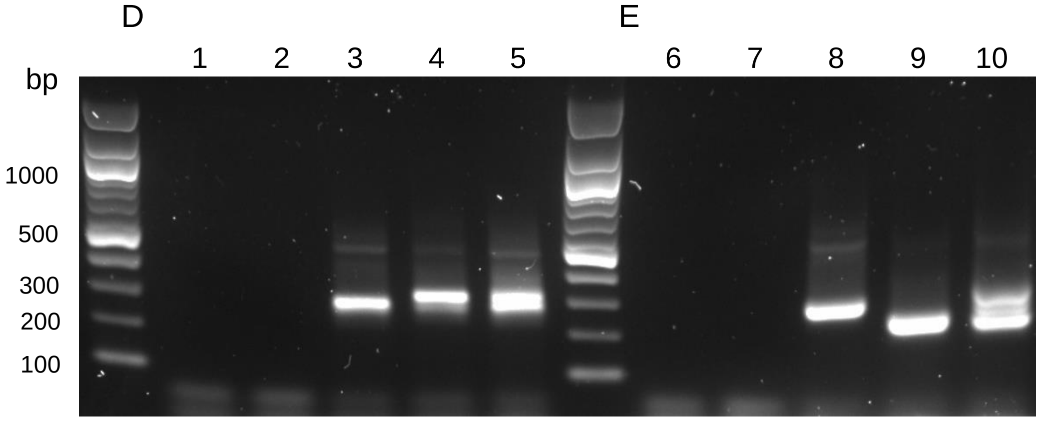

Figures D and E. Three-primer duplex COMPAS-PCR for *S. trutta*, *S. salar* and hybrids using primers 5SNTS-23F-W1 + 5SNTS-23R+3-mamT + 5SNTS-23R+3-mamG-m2 (278 & 300bp, A) and 5SNTS-23F-W1 + 5SNTS-23R+3-mamT 5SNTS-21R+57 (278 & 225bp, B). Lanes 1 & 6: H_2O PCR negative control; lanes 2 & 7: *Oncorhynchus tshawytscha* method negative control; lanes 3 & 8: *S. salar* S4826; lanes 4 & 9: *S. trutta* T4816; and lanes 5 & 10: *S. salar* x *S. trutta* hybrid ST4819.
